# Resting and Post Bronchial Challenge Testing Carbon Dioxide Partial Pressure in Individuals with and without Asthma

**DOI:** 10.1371/journal.pone.0032464

**Published:** 2012-03-07

**Authors:** David Miedinger, Anja Jochmann, Lucia Schoenenberger, Prashant N. Chhajed, Jörg D. Leuppi

**Affiliations:** Internal Medicine, University Hospital, Basel, Switzerland; Abramson Research Center, United States of America

## Abstract

**Objective:**

There is conflicting evidence about resting carbon dioxide levels in asthmatic individuals. We wanted to determine if transcutaneously measured carbon dioxide levels prior and during bronchial provocation testing differ according to asthma status reflecting dysfunctional breathing.

**Methods:**

We investigated active firefighters and policemen by means of a validated questionnaire on respiratory symptoms, spirometry, bronchial challenge testing with methacholine (MCT) and measurement of transcutaneous blood carbon dioxide partial pressure (PtcCO_2_) at rest prior performing spirometry, one minute and five minutes after termination of MCT. A respiratory physician blinded to the PtcCO_2_ results assigned a diagnosis of asthma after reviewing the available study data and the files of the workers medical screening program.

**Results:**

The study sample consisted of 128 male and 10 female individuals. Fifteen individuals (11%) had physician-diagnosed asthma. There was no clinically important difference in median PtcCO_2_ at rest, one and five minutes after recovery from MCT in asthmatics compared to non-asthmatics (35.6 vs 35.7 mmHg, p = 0.466; 34.7 vs 33.4 mmHg, p = 0.245 and 37.4 vs 36.4 mmHg, p = 0.732). The median drop in PtcCO_2_ during MCT and the increase after MCT was lower in asthmatics compared to non-asthmatics (0.1 vs 3.2 mmHg, p = 0.014 and 1.9 vs 2.9 mmHg, p = 0.025).

**Conclusions:**

PtcCO_2_ levels at rest prior and during recovery after MCT do not differ in individuals with or without physician diagnosed asthma. The fall and subsequent increase in PtcCO_2_ levels are higher in non-asthmatics than in asthmatics and seems to be related with increased number of respiratory maneuvers during MCT.

## Introduction

Asthma is associated with a chronic inflammation of the airways associated with symptoms such as cough, wheezing, sputum production and chest tightness. Further several studies have shown an association between asthma and higher anxiety sensitivity scores and panic disorder [1], [2], [3].

Breathing abnormalities such as an abnormal breathing pattern, unsteadiness of breathing in response to exercise or voluntary hyperventilation, increased respiratory rate, a predominantly intercostal respiratory effort as well as frequent sighing have been classified as dysfunctional breathing [4]. Thomas and co-workers have shown that dysfunctional breathing can be found in a third of asthmatic woman and a fifth of asthmatic men seen in general practice and in about 8% of the general population when evaluated with a questionnaire to detect functional breathing problems, the Nijmwegen Questionnaire [4], [5]. The assumption that dysfunctional breathing might be associated with lower end-tidal carbon dioxide partial pressures (PETCO_2_) has led to the idea to offer asthmatics breathing retraining to raise PETCO_2_ [6]. It was shown in an experimental setting that increasing PETCO_2_ can decrease respiratory resistance while decreasing PETCO_2_ in turn led to an increase in resistance and decrease in airway reactance in asthmatic individuals [7]. Currently, there is conflicting data about resting and post bronchial challenge testing PETCO_2_ and their association with asthma status. Therefore we wanted to determine prospectively, if resting transcutaneous blood carbon dioxide partial pressures (PtCO_2_) prior and after bronchial challenge testing or the change in PtcCO_2_ were different in individuals with or without asthma.

## Materials and Methods

The study was approved by the local ethics committee EKBB (Ethikkommission beider Basel) and all participants gave written informed consent.

**Figure 1:**
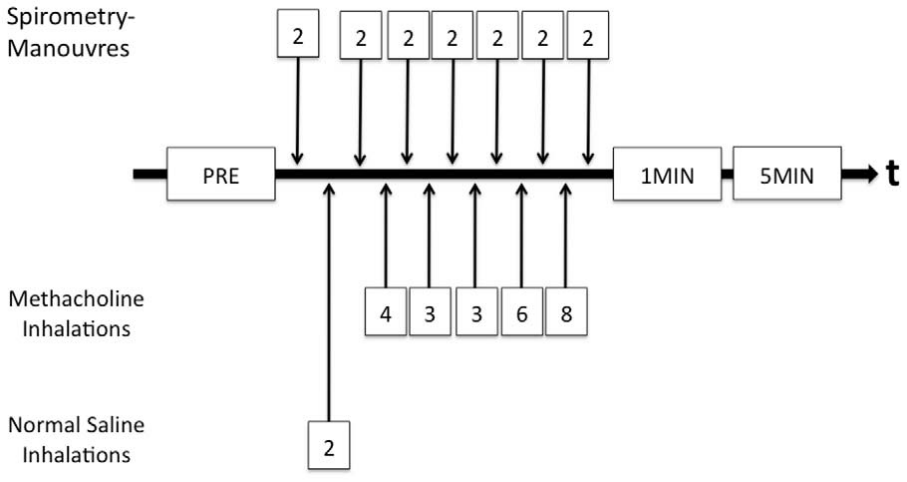
Sequence of PtcCO_2_ measurements, inhalation maneuvers and spirometry. PRE = transcutaneous blood carbon dioxide partial pressures (PtcCO_2_) prior to methacholine bronchial challenge test (MCT), 1MIN = PtcCO_2_ one minute after termination of MCT, 5MIN = PtcCO_2_ five minutes after termination of MCT.

In the context of a study in active firefighters and policemen in Switzerland to measure asthma prevalence, workers answered a validated questionnaire on respiratory symptoms. The population studied was a convenience sample of workers recruited among the current firefighting and police workforce in the city of Basel. Subsequently, they underwent bronchoprovocation testing with methacholine (MCT) according to a widely applied standardized protocol, that has been used by the European Community Respiratory Health Survey (ECRHS) [8]. The study protocol included a defined sequence of inhalations with a maximum cumulative dose of 2 mg methacholine and spirometry maneuvers (Figure 1). The test was considered positive when an individual FEV1 fell by 20% or greater during MCT. We allowed spontaneous recovery of lung function in all individuals for five minutes after all inhalation steps were performed or the test was considered positive before a short acting bronchodilatator was eventually administered.

During the metacholine challenge, PtcCO_2_ was measured by using a validated, combined ear sensor for pulse oximetry and PtcCO_2_ (SenTec AG, Therwil, Switzerland), permitting to collect data non-invasively and corresponding to ABG analysis [9]. The response time of the sensor regarding measurement of PtcCO_2_ was shown to be less than one minute [10]. The change of the sensor membrane, the device calibration and sensor application was done as required by the manufacturer. Patients were seated and the sensor was taken out of the docking station immediately before the measurement was started. We recorded PtcCO_2_ of all subjects at rest prior (*PRE*), one and five minutes after (*1MIN* and *5MIN*) finishing MCT. Recording of PtcCO_2_ was always performed prior to performing spirometry. For the first reading (*PRE*) we followed the manufacturer's recommendation to wait until the “PCO_2_ stabilizing” message disappeared from the monitor.

Sensitivity analysis was performed by assigning individuals a diagnosis of asthma, according to two widely used definitions: Having a) *physician-diagnosed asthma* or b) had wheezing in the last twelve months plus a positive result in the MCH test (*Wheeze/MCHPD20+*) [11]). A respiratory physician blinded to the PtcCO_2_ results obtained assigned a diagnosis of asthma (*physician-diagnosed asthma*), after reviewing the available data (questionnaire, lung function, methacholine and exhaled nitric oxide test results, medication use and files of workers the medical screening program). For comparison we also present the data of those individuals in which the MCT was considered positive, when a drop in FEV1 of 20% or greater occurred during the test (*MCHPD20+*).

Continuous variables were expressed as median and interquartile range. Non-parametric tests were performed for paired and unpaired measures as most of the data was not normally distributed. Statistic tests were performed using SPSS-software (version 19, IBM Corporation, Somers, New York, USA).

## Results

We investigated 128 male and 10 female individuals with a mean age of 40 years (range 22–63 years). Twenty-four individuals (17%) had a positive MCT (*MCHPD20+*), 15 (11%) had *physician-diagnosed asthma*, and 12 (9%) had wheeze in the last 12 months and had a positive MCT (*Wheeze/MCHPD20+*).

Median PtcCO_2_ values regardless of asthma status measured prior and during MCT can be seen in Table 1. The median PtcCO_2_ decrease during MCT (*PRE-1MIN*) was 3.0 mmHg (IQR 4.7 mmHg) whereas the median increase during recovery phase after MCT (1MIN-5MIN) was 2.7 mmHg (IQR 3.4). Median PtcCO_2_ five minutes after the end of MCT, was not clinically or statistically significantly different from pre-test values (PRE-5MIN, p = 0.108).

**Table 1:** Median values of transcutaneous carbon dioxide partial pressures (PtcCO_2_) during the course of bronchial challenge testing with methacholine.

	n	PtcCO_2_ (mmHg)	PtcCO_2_ (mmHg)	p-value
PRE-1MIN	138	PRE 35.6 (5.5)	1MIN 33.5 (5.7)	<0.001
PRE-5MIN	138	PRE 35.6 (5.5)	5MIN 36.4 (5.0)	0.108
1MIN–5MIN	138	1MIN 33.5 (5.7)	5MIN 36.4 (5.0)	<0.001

Data is expressed as median (interquartile range). PtcCO_2_ = transcutaneous blood carbon dioxide partial pressure. PRE = PtcCO_2_ prior to methacholine bronchial challenge, 1MIN = PtcCO_2_ one minute after termination of methacholine bronchial challenge, 5MIN = PtcCO_2_ five minutes after termination of methacholine bronchial challenge.

Sensitivity analysis for the different asthma diagnosis revealed no significant differences in median PtcCO_2_, prior, during and after recovery from MCT between the asthmatic and non-asthmatic individuals for all three definitions (Table 2). However when comparing the median change in individuals PtcCO_2_ during MCT those classified as having asthma had lower fall than those without asthma, accordingly during recovery phase after the test the increase in PtcCO_2_ was higher in non-asthmatics compared to asthmatics (Table 3).

**Table 2:** Comparison of median transcutaneous carbon dioxide partial pressures (PtcCO_2_) according to asthma definition during the course of bronchial challenge testing with methacholine.

			PtcCO_2_(mmHg)		
Asthmadefinition		n	PRE	1 Min	5 Min
*Physician-* *diagnosed*	Present	15	35.6 (4.5)^a^	34.7 (6.5)^a^	37.4 (5.4)^a^
	Not present	123	35.7 (5.5)	33.4 (5.9)	36.4 (4.9)
*Wheeze/MCHPD20*	Present	12	36.3 (6.0)^a^	35.1 (5.6)^a^	37.5 (5.0)^a^
	Not present	126	35.6 (5.5)	33.4 (5.9)	36.4 (5.0)
*MCHPD20*	Present	24	35.7 (5.3)^a^	34.9 (6.1)^b^	37.2 (4.2)^a^
	Not present	114	35.6 (5.6)	33.2 (5.8)	36.4 (5.2)

Data is expressed as median (interquartile range). PtcCO_2_ = transcutaneous blood carbon dioxide partial pressures. PRE = PtcCO_2_ prior to methacholine bronchial challenge, 1MIN = PtcCO_2_ one minute after termination of methacholine bronchial challenge, 5MIN = PtcCO_2_ five minutes after termination of methacholine bronchial challenge. *MCHPD20* = Fall in FEV1 ≥20% during bronchial challenge testing with methacholine.

a = Not significant with p>0.1,

b = Not significant with p<0.1.

**Table 3:** Comparison of median values of change of transcutaneous carbon dioxide partial pressures (PtcCO_2_) according to asthma definition during testing and during recovery after methacholine provocation testing.

			Δ PtcCO_2_(mmHg)		
Asthmadefinition		n	PRE-1MIN	PRE-5MIN	1MIN–5MIN
*Physician-* *diagnosed*	Present	15	0.1 (4.3)*	−1.4 (3.4)^a^	−1.9 (1.9)*
	Not present	123	3.2 (5.0)	−.6 (4.0)	−2.9 (3.6)
*Wheeze/MCHPD20*	Present	12	0.6 (4.0)*	−1.2 (3.1)^a^	−1.8 (3.0)^b^
	Not present	126	3.2 (4.8)	−0.7 (3.9)	−2.8 (3.5)
*MCHPD20*	Present	24	1.0 (4.2)*	−1.0 (3.3)^a^	−1.8 (2.9)*
	Not present	114	3.2 (5.2)	−0.7 (4.0)	−2.9 (3.5)

Data is expressed as median (interquartile range). PtcCO_2_ = transcutaneous blood carbon dioxide partial pressures. PRE = PtcCO_2_ prior to methacholine bronchial challenge, 1MIN = PtcCO_2_ one minute after termination of methacholine bronchial challenge, 5MIN = PtcCO_2_ five minutes after termination of methacholine bronchial challenge. *MCHPD20* = Fall in FEV1 ≥20% during bronchial challenge testing with methacholine.

a = Not significant with p>0.1,

b = Not significant with p<0.1,

* = Significant with p≤0.05.

## Discussion

There was no clinically or statistically important difference in median PtcCO_2_ in individuals with asthma at rest, immediately after and after recovery from MCT. However the change in PtcCO_2_ during the MCT was more pronounced in individuals without asthma, most likely because they performed more forced in- and expiratory maneuvers during MCT resulting in more iatrogenic induced hyperventilation.

Firefighters and policemen are exposed to various airway irritants during work and have been shown to have a high prevalence of respiratory symptoms and asthma [12], [13]. Therefore the study was conducted to measure and compare the prevalence of respiratory symptoms, bronchial hyperresponsiveness and asthma in these populations. While it was possible to measure over 90% of the active professional firefighters in the city of Basel, we had to randomly select a convenience sample of 60 out of about 600 policemen in the active workforce of the city.

In our study population we did not find clinically nor statistically significant difference in resting and post MCT PtcCO_2_ levels according to asthma status. This finding is in discordance with several studies that have reported resting PETCO2 levels to be lower in asthmatic individuals [7], [14], [15]. One must be cautious when comparing these findings with our results. While the patients in aforementioned studies were recruited among a hospital population, we have identified asthmatics in a population sample of apparent healthy workers. Dysfunctional breathing has been reported to induce respiratory symptoms [16] and this may lead asthmatics to seek medical treatment. Thus, the prevalence of dysfunctional breathing can be expected to be lower in asthmatics identified during studies at the workplace or in population studies.

Forced inspiratory and expiratory maneuvers during MCT will reduce rest carbon dioxide levels in the blood [16]. The device we used to measure PtcCO_2_ has been validated and was shown to correlate well with invasive determination of blood CO2 levels [9]. Further this device has successfully been used to diagnose hyperventilation syndrome in individual patients in the clinic [17]. Performing MCT has been shown to induce transient hypoxemia post testing as well as to induce ventilation/perfusion mismatch [18], [19]. In our study we could see a more pronounced drop in PtcCO_2_ in individuals who had a negative MCT and were not diagnosed with asthma. Healthy individuals with light or no bronchial hyperresponsiveness to methacholine need to perform more inhalation maneuvers to inhale increasing methacholine doses during the MCT. As spirometry is performed after each dose step, the number of spirometries increases accordingly. Koulouris and coworkers suggested that performing bronchial challenge testing in asthmatics may lead to an increase of dead space [20] which would lead to less CO2 elimination. However as a group the median PtcCO_2_ levels were not clinically or statistically different according to asthma status one minute and five minutes after termination of MCH when spontaneous recovery was allowed.

Dysfunctional breathing might be a temporal rather than a continuous phenomenon and thus the measurements and bronchial challenge test we performed at a single occasion were unable to detect hypocapnia as a result of dysfunctional breathing in the study participants. To the authors knowledge there are no data regarding the intra-individual variability of PETCO2 or PtcCO_2_ in asthmatics. Using the Nijmegen questionnaire to determine hyperventilation-associated complaints would have allowed us to correlate these findings with PtcCO_2_ at rest and during bronchial challenge testing. However there has been criticism that the Nijmwegen questionnaire is not a valid tool for identification of hyperventilation in asthmatics because of the co-occurrence of asthma and anxiety - in which the symptoms might be similar – and due to the fact that this questionnaire was validated only in a non-asthmatic population [21], [22].

A spontaneous fall in PETCO2 of at least 0.25% during the adaptation period before a hyperventilation provocation test was shown to have an accuracy of 70% for the diagnosis of hyperventilation syndrome, while the responses of PETCO2 during provocation test were reported not to be useful for the diagnosis of hyperventilation syndrome [23]. Another reason for lack of difference in resting and post MCH levels might be the fact that our sample consisted of mainly male participants. In Switzerland, firefighting and less so policing is still a male dominated area. As shown in asthmatics and non-asthmatics dysfunctional breathing seems to be more common in female than in males [5] and this increases the risk of a type II error due to insufficient power.

### Conclusions

In a group of workers undergoing bronchial challenge testing to diagnose asthma we could not find an association of low resting PtcCO_2_ levels with asthma status reflecting chronic hyperventilation or dysfunctional breathing. Median PtcCO_2_ levels and change in PtcCO_2_ levels during bronchial challenge testing with methacholine is dependent on the number of respiratory maneuvers during the test. PtcCO_2_ levels during recovery or five minutes after bronchial challenge testing do not help to identify individuals with or without asthma. Further studies are needed to determine the association of individual resting PtcCO_2_ levels with dysfunctional breathing and if the breathing pattern and thus PtcCO_2_ levels are different regarding the presence of dysfunctional breathing during experimental nonspecific bronchial challenge.
